# Cultural awareness scale: psychometric properties and applicability in assessing cultural competence among polish nursing students

**DOI:** 10.1186/s12912-025-03181-y

**Published:** 2025-05-15

**Authors:** Ilona Cieślak, Mariusz Panczyk, Joanna Gotlib-Małkowska, Mariusz Jaworski

**Affiliations:** Department of Education and Research in Health Sciences, Faculty of Health Sciences, Medical University of Warsaw, Warsaw, Poland

**Keywords:** Cultural competence, Nursing education, Psychometric validation, Cultural awareness scale (CAS), Intercultural communication, Nursing students

## Abstract

**Background:**

Cultural competence is an essential skill for nursing students to provide effective care in multicultural healthcare settings. Despite the availability of various tools globally, there are limited number of validated instruments for assessing cultural awareness among nursing students in Poland. This study aimed to culturally and linguistically adapt the Cultural Awareness Scale (CAS) to the Polish context and evaluate its psychometric properties.

**Materials and methods:**

A cross-sectional, web-based survey was conducted between May and June 2024 among 1,020 nursing students from nine Polish medical universities. The CAS was translated and adapted following WHO guidelines for cultural and linguistic adaptation. Psychometric evaluation included exploratory and confirmatory factor analyses (EFA, CFA), reliability testing using Cronbach’s alpha and McDonald’s omega, and assessments of validity, including convergent and known-groups validity.

**Results:**

The Polish version of the CAS (CAS_P) demonstrated high reliability, with a Cronbach’s alpha of 0.892 and McDonald’s omega of 0.908. EFA confirmed the multidimensional structure of the scale, while CFA indicated moderate model fit (CFI = 0.797, TLI = 0.781, RMSEA = 0.0735). Convergent validity analysis showed significant correlations between CAS domains and personality traits such as altruism and openness to experience (*p* < 0.001). Known-groups validity analysis revealed that nursing students with prior intercultural education scored significantly higher on all CAS domains (*p* < 0.05), highlighting the impact of formal training on cultural awareness. The Behaviors/Comfort with Interactions subscale showed lower reliability (Cronbach’s alpha = 0.592), suggesting cultural-specific variations in responses.

**Conclusions:**

The CAS_P is a reliable and valid instrument for assessing cultural awareness among Polish nursing students. Its implementation can guide curriculum development and enhance intercultural competence in nursing education. Further refinements are necessary to improve the scale’s sensitivity to local cultural contexts.

**Clinical trial number:**

Not applicable.

**Supplementary Information:**

The online version contains supplementary material available at 10.1186/s12912-025-03181-y.

## Introduction

The development of cultural competence during nursing studies is essential to prepare future healthcare professionals for the challenges of work in multicultural settings [1]. In a globalized world, nurses regularly provide care to patients who have different values, beliefs and health practices [2]. Cultural diversity in healthcare presents nursing students with challenges that require not only medical knowledge, but also interpersonal skills and sensitivity to cultural differences. Patients may have different perspectives on diagnosis, treatment and even the very concept of health and illness [3]. Therefore, future nurses need to be able to adapt their professional practice to meet the specific needs of individual patients, taking into account their cultural background [4].

Successful development of cultural competence in nursing students requires a reliable framework and standardized research tools to be used for the description of this skill [5]. Only through the identification of student strengths and weaknesses can the curriculum be adapted to student real needs. By assessing different aspects of cultural competence, these tools provide objective data to inform curriculum modifications [6]. Regular monitoring of the level of these competences makes it possible to track student progress throughout their education, which in turn facilitates targeted improvement interventions [7]. To ensure optimal implementation, these tools should be tailored to suit a given educational context.

In Poland, nursing education follows the guidelines of Directive 2015/55/EU and incorporates standards including, among others, issues related to cultural and religious diversity and anti-discrimination [8]. Although universities have autonomy in constructing curricula, they should base them on the tenets of evidence-based education. Content related to developing cultural competence is being incorporated both as separate subjects and within theoretical classes and clinical practices. However, systematic analyses of the effectiveness of these activities are lacking, which makes it difficult to assess their real impact on preparing students to work in a culturally diverse environment.

There are several research tools designed to assess cultural competence in nursing students, e.g. Transcultural Self-Efficacy Tool (TSET) [9], Cultural Awareness Scale (CAS) [10], or Inventory for assessing the process of cultural competence among health care professionals - student version [11]. To the best of our knowledge, no tool designed to measure cultural competence in nursing students has yet been validated or culturally or linguistically adapted in Poland.

Therefore, in the present study, we aimed to validate and linguistically and culturally adapt the Cultural Awareness Scale. The choice of the research tool under consideration has been determined by several factors. With the author’s consent, the CAS is a free-of-charge scale with very good psychometric properties. It has also been successfully validated in several countries around the world, including South Korea [12], Cyprus [13] and Slovenia [14]. In addition, the CAS measures the aspects of cultural competence not found in other tools, e.g. the Research Issues subscale.

## Background

The process of social change in Poland, including a steady rise in migration, began in 2014 with Russia’s first aggression against eastern Ukraine (Donbas). Since then, the number of foreigners arriving in Poland has steadily increased each year, reaching approximately 457,200 individuals in 2020 and almost 1.5 million in 2021 [15]. A major refugee crisis was triggered by Russia’s full-scale invasion of Ukraine on 24 February 2022, which resulted in the arrival of 7.8 million refugees into EU countries, of which more than 3 million found their way to Poland and hundreds of thousands more to Romania (over 880,000) and Hungary (over 568,000). There are currently over 981,000 refugees from Ukraine in Poland [16]. Due to the lack of systematic and ongoing updating of migration data in Poland, a precise determination of the number of foreigners residing in the country remains impossible. Nevertheless, according to estimates by the Social Insurance Institution, nearly 2.5 million migrants resided in Poland in 2024, and 1.5 million of them were covered by social and health insurance, entitling them to benefits offered by the health care system. Poland has experienced a significant increase in the number of foreigners residing and working within its borders [17]. According to Statistics Poland (GUS), as of May 2024, approximately 1,024,200 foreigners were employed in the country, accounting for 6.7% of the total workforce. These individuals hailed from over 150 countries, with the largest groups originating from Ukraine, Belarus, Georgia, India, Nepal, Bangladesh, Uzbekistan, and the Philippines. This growing multicultural population presents new challenges and opportunities for the Polish healthcare system, particularly in nursing, where culturally competent care is essential for effective patient outcomes [18].

Foreigners arriving in Poland include those who wish to pursue education, including in the medical and health sciences (e.g. nursing). In the academic year 2022/2023, there were more than 102,000 foreign students at Polish universities (including more than 48,000 from Ukraine and 12,000 from Belarus), compared to only 42,000 in 2014 [19]. Increased intercultural diversity requires students to collaborate in multicultural groups. For future health professionals who will encounter patients and colleagues from different cultures in their professional practice, such skills are of particular importance.

## Aim

The study aimed to assess the validity and reliability of the Cultural Awareness Scale on a sample of Polish nursing students.

## Materials and methods

### Design and setting

This cross-sectional, web-based national survey was conducted between May and June 2024, targeting all twelve Polish medical universities offering bachelor’s and master’s degree programs in nursing. Of these, nine universities participated, involving students enrolled in their bachelor and master nursing programs.

### Sample size

The study population consisted of all students enrolled in bachelor and master nursing programs at nine of the twelve medical universities in Poland that were invited to participate, amounting to a potential respondent pool of 7,000 individuals. Each participating university appointed a coordinator responsible for collecting the surveys at their institution. Ultimately, 1,020 fully completed questionnaires were obtained. This response rate allowed for robust data analysis, with a calculated margin of error of 2.84% at a 95% confidence interval, assuming a proportion of 0.5.

### Participants

The sample comprised a total of 1020 nursing students (Table 1). The age distribution of the participants was 26.4 ± 7.6 years, ranging from 19 to 61 years. Regarding their level of study, 51.08% (*n* = 521) were in their first-cycle studies and 48.92% (*n* = 499) in their second-cycle studies. The sample was predominantly female (90.1%, *n* = 919). The students were enrolled in various medical universities across Poland.

**Table 1:** Sample characteristics

Variable	*N*	%
University		
Medical University of Białystok	386	37.84
Medical University of Warsaw	348	34.12
Medical University of Lublin	129	12.65
Pomeranian Medical University	57	5.59
Poznan University of Medical Sciences	33	3.24
Medical University of Silesia	15	1.47
Jagiellonian University Medical College	10	0.98
Medical University of Gdańsk	7	0.69
Jan Kochanowski University of Kielce	1	0.10
Other universities	34	3.33
Program		
First-cycle studies	521	51.08
Second-cycle studies	499	48.92
Gender		
Female	919	90.10
Male	93	9.12
Non-binary	5	0.49
Other/ Refusal to answer	3	0.29

### Ethical consideration

This study was performed in accordance with the Declaration of Helsinki. The study protocol was approved by the University Bioethics Committee (IRB approval No. AKBE/319/2023). Participants were informed of confidentiality measures prior to data collection to ensure that no personal, sensitive or IP address information was recorded and strict anonymity was maintained throughout the study. Due to the large number of subjects, the nature of the study (multi-centre, non-invasive and non-interventional) and the assurance of anonymity during the data collection process, participants gave verbal consent to participate in the study. Prior to the start of the study, they were informed of the anonymity and confidentiality rules in place. The data that were subsequently collected, statistically analysed and presented in this article do not identify individual participants.

### Instrument

The Cultural Awareness Scale (CAS) was developed in 2003 by Rew et al. [10] to measure cultural awareness in nursing students. The development of the Cultural Awareness Scale (CAS) was grounded in the theoretical framework of cultural competence proposed by Campinha-Bacote, which conceptualizes cultural competence as a dynamic and ongoing process rather than a static end-state. This model identifies five interrelated components: cultural awareness, cultural knowledge, cultural skill, cultural encounters, and cultural desire. The CAS specifically operationalizes the component of cultural awareness, focusing on the cognitive, affective, and behavioral dimensions of how nursing students perceive and interact with culturally diverse populations. The scale items were derived from an extensive review of literature on nursing education and transcultural care and were designed to reflect the educational, interpersonal, and clinical aspects of cultural awareness development [10]. The CAS consists of 36 items derived from a literature review on cultural awareness, sensitivity, and competence in nursing. Respondents rate their agreement with each statement using a 7-point Likert scale, ranging from “strongly disagree” to “strongly agree”. In the studies by Rew et al. [10], the Cronbach’s alpha coefficient for the entire scale was 0.82, and factor analysis confirmed its multidimensional structure, with five factors explaining 51% of the variance. The authors of the current study obtained written permission from the author of the CAS to validate and adapt it to the Polish context.

The CAS assesses five key domains of cultural awareness in nursing education, each reflecting a different aspect of student experiences and attitudes towards cultural competence. Table 2 provides a detailed description of each CAS domain.

**Table 2:** CAS domains with their characteristics, a sample item and number of items per domain [10]

CAS subscale	Number of items	Characteristics	Sample item
General Educational Experience (GEE)	13	Items assess how well nursing education at a given university addresses cultural diversity and measure the impact of the educational setting on students’ cultural awareness.	The instructors at this nursing school adequately address multicultural issues in nursing
Cognitive Awareness (CA)	9	Items assess student understanding of how culture influences beliefs, attitudes and behaviors, both their own and those of others, and reflect their awareness of cultural diversity at a cognitive level.	I think my behaviors are influenced by my culture
Research Issues (RI)	4	Items assess student awareness of and engagement with cultural diversity in nursing research.	The faculty at this school of nursing conducts research that considers multicultural aspects of health-related issues
Behaviors/Comfort with Interactions (CI)	6	Items assess students’ self-reported comfort and behaviors when interacting with people from different cultural backgrounds. They include statements related to comfort levels in intercultural interactions.	I feel comfortable working with patients of all ethnic groups
Patient Care/Clinical Issues (CP)	4	Items represent student attitudes and behaviors related to providing culturally competent clinical care. They assess the willingness and desire to respect and accommodate cultural diversity in patient care.	I respect the decisions of my patients when they are influenced by their culture, even if I disagree

### Research procedure

The validation process of the CAS was comprehensive and multi-faceted, incorporating both linguistic and cultural adaptation, followed by an in-depth assessment of its psychometric properties. The initial phase, following the guidelines of Sousa, Rojjanasrirat [20] and the World Health Organization “Process of Translation and Adaptation of Instruments” [21], aimed to ensure relevance and comprehensibility of the scale within the Polish context.

### Language validity

The study used a dual translation method for cultural and linguistic adaptation of the CAS. First, the scale was translated from English into Polish by a professional translator, a native English speaker trained in teaching medical and health sciences. Subsequent evaluation involved a panel of expert reviewers who assessed the comprehensibility of each item and the applicability of the Polish version of the CAS (CAS_P) for measuring cultural competence among Polish nursing students. Content validity was rigorously assessed by calculating both the Item-Level Content Validity Index (I-CVI) and the Scale-Level Content Validity Index (S-CVI), based on the methodology proposed by Polit, Beck, Owen [22].

The expert panel, comprising seven experts from diverse academic disciplines including medical sciences, health sciences, sociology, psychology, statistics, and academia, rated each item using a 4-point Likert scale: 1 (not relevant), 2 (somewhat relevant), 3 (quite relevant), and 4 (highly relevant). A CVI greater than 0.80 was considered to indicate satisfactory content validity.

Following consensus among the study authors and the expert reviewers, a second translator, not previously involved in CAS, performed a back-translation from Polish into English. The back-translated version was then reviewed by Rew, the author of the CAS. Based on her feedback, a multidisciplinary team analyzed the cross-cultural equivalence between the original and the Polish version, CAS_P. This team included a statistical validation specialist, all research team members, and both translators involved in the initial stages.

The team reached a consensus and developed the CAS_P version, which was used for preliminary testing. The Polish CAS_P was first tested on a random sample of 10 nursing students from the Medical University of Warsaw, which confirmed the comprehensibility of all questions, although some minor lexical adjustments were made. After reaching a consensus, the final version of CAS_P was prepared for the pilot study.

### Pilot study

Out of 1,014 nursing students invited from the Medical University of Warsaw, 74 voluntarily completed the questionnaire during the one-week pilot phase. No reminders were sent, and participation was entirely voluntary and anonymous. After completing the survey, semi-structured interviews were conducted with 15 students who agreed to discuss potentially unclear or culturally ambiguous statements. The list of questions posed to the nursing students can be found in Supplementary 1. The pilot study served a critical role in evaluating the comprehensibility and cultural appropriateness of the CAS_P items. Based on the pilot results and feedback from the expert panel, one item was removed due to persistent misinterpretation, and two negatively worded statements were rephrased for clarity and alignment with Polish cultural norms. Additionally, minor lexical changes were introduced in several items to enhance clarity without altering their original meaning. These changes were implemented before launching the main study, resulting in a refined and culturally adapted final version of the CAS_P.

### Main study

The main objective of the study was to evaluate the psychometric properties of the CAS_P adapted to the Polish context. The questionnaire was administered online using the Lime Survey platform. Coordinators from the participating universities received a survey link, which they forwarded to all nursing students at their institutions. To maximize response rates, the survey link was sent to students on three separate occasions.

### Psychometric properties

The psychometric properties of the CAS were evaluated using a structured approach, focusing on reliability and validity.

### Pre-analysis screening

All 36 items of the Cultural Attitudes Scale (CAS) employ a sevenpoint Likert format and were therefore treated as *ordered categorical*. Descriptive statistics (means, standard deviations, skewness, kurtosis) were inspected to confirm that floor and ceiling effects were below the 1% criterion recommended for healthoutcome measures [23].

### Assessment of factorability

Polychoric correlations were preferred over Pearson’s coefficients because they yield less biased estimates for ordinal items with potential nonnormality [24]. Sampling adequacy was verified with the KaiserMeyerOlkin (KMO) index and Bartlett’s test of sphericity; both procedures are prerequisites for factor analysis [25].

### Dimensionality determination

The number of underlying factors was established with the optimal implementation of parallel analysis for ordered items [26]. Parallel analysis is widely regarded as the most accurate empirical criterion for deciding dimensionality, outperforming the Kaisereigenvalue rule and the scree plot, particularly in ordinal data sets.

### Exploratory factor analysis (EFA)

EFA was performed on the polychoric correlation matrix using robust unweighted leastsquares (RULS) extraction, followed by Direct Oblimin rotation (γ = 0). RULS provides consistent parameter estimates under violations of multivariate normality and, together with polychoric input, is recommended for Likerttype variables [27]. To evaluate stability, the sample was split into two equivalent halves with the Solomon procedure, and the factor structure was replicated in each subsample. Items were assigned to a factor when their primary loading was ≥ 0.40 and exceeded secondary loadings by at least 0.10. Reliability and precision of latent scores were judged with the Overall Reliability of fullyInformative prior Oblique NEAP scores (ORION) and the Factor Determinacy Index (FDI), which quantify the quality of factor score estimation in oblique solutions [28].

### Confirmatory factor analysis (CFA)

The EFAderived structure was crossvalidated via mildrestricted CFA. This twostep leastsquares approach first estimates all free loadings with a datadriven algorithm (MORGANA) and then imposes theoretically motivated zero constraints.

- Parameterisation and identification. Latent variances were fixed to 1, primary loadings were freely estimated, and crossloadings as well as error covariances were fixed to 0, yielding an overidentified model that satisfies the t–s rule.
- Estimator and test statistic. Leastsquares estimation on the polychoric matrix was paired with the LOSEFER scaling of the χ² statistic, which controls for Type I error inflation in categorical indicators [29].
- Modelfit evaluation. Global fit was judged using multiple indices recommended by Hu and Bentler [30] and Steiger [31]: χ²/df, rootmeansquare error of approximation (RMSEA), comparative fit index (CFI), standardised rootmeansquare residual (SRMR), and the Tucker Lewis Index (TLI). Local fit was assessed through standardised residuals and modification indices; parameters were freed only when both statistically and theoretically justified. Biascorrected and accelerated (BCa) bootstrap confidence intervals were calculated for all factor loadings to confirm their statistical significance.

### Reliability

Internal consistency was estimated with Cronbach’s α and McDonald’s ω, with ≥ 0.70 considered acceptable for research instruments [32].

### Validity evidence

Three subscales of the NEO Personality Inventory-Revised (NEO-PI-R), originally developed by Costa and McCrae [33] and adapted into Polish by Siuta [34], were used to assess the convergent validity of the CAS. Correlation analysis using Pearson’s correlation coefficient was conducted to assess relationships with CAS domains.

The NEO-PI-R is a psychometric personality questionnaire designed to assess five major personality factors (Neuroticism, Extraversion, Openness to Experience, Agreeableness, and Conscientiousness)—and their 30 facets, providing a comprehensive view of personality. It consists of 240 items rated on a five-point Likert scale ranging from ‘strongly disagree’ to ‘strongly agree’. For this study, the Ideas subscale of Openness to Experience, the Altruism subscale of Agreeableness, and the Warmth subscale of Extraversion were selected [33].

We focused on these subscales because they provide precise insights into traits that directly impact social interactions and openness to cultural diversity, specifically reflecting characteristics essential for effective intercultural engagement. Compared to broader Big Five domains, these subscales capture subtle variations in behaviours and attitudes that may be missed by broader assessments, making them more relevant for evaluating cultural competence. This approach allows for a deeper understanding of how specific traits influence attitudes toward cultural diversity and intercultural interactions that are crucial to the development of cultural competence.

The Ideas subscale assesses openness to new ideas and diverse beliefs, e.g. openness to differing worldviews, which facilitates cultural understanding and acceptance of other cultures. The Warmth subscale reflects positive social attitudes and ease in forming relationships, which facilitates effective and empathetic intercultural communication, fostering mutual understanding. The Altruism subscale measures empathy and a willingness to help others, such as demonstrating care and support in intercultural interactions, which are essential for engaging positively with individuals from diverse cultural backgrounds [33].

The research instrument included two special questions designed to assess known-groups validity for all domains of the CAS: (1) Are you open to learning about new cultures? and (2) Have you ever participated in activities (e.g. lectures, training courses, workshops, exercises, seminars) on intercultural communication? To assess known-groups validity, One-Way ANOVA was used to analyze mean differences. The Games-Howell Post-Hoc Test was employed to compare all possible combinations of group differences.

### Software

All factor analyses were conducted in FACTOR 12.06.07 (Rovira i Virgili University). Descriptive statistics, reliability coefficients, and validity tests were carried out in STATISTICA 13.3 and crosschecked in Mplus 6.12. The significance level for all inferential tests was set at α = 0.05 (twotailed).

## Results

### Pilot study

The pilot study results analysis showed that the reliability of the CAS was α = 0.737 and the McDonald’s coefficient ω was 0.789, with seven items negatively correlating with the overall scale (Table 1A, see Supplementary 2). As a follow-up to the pilot study, the same panel of experts, together with the authors of the study, reassessed the cultural and linguistic validity of the translated instrument. Through discussion, because of the negative correlation with the scale’s total score and because of the cultural context, a decision was made to remove one item from the final version of the CAS: “My nursing instructors seem interested in learning how their classroom behaviors may discourage students from certain cultural or ethnic groups”. Two items were reversed: “When I have an opportunity to help someone, I am less likely to offer help to people from other cultural backgrounds” and “I am less patient with individuals from certain cultural backgrounds” (the wording of the items has been changed to: “When I have the opportunity to help someone, I offer assistance more often to individuals from my cultural background”, “I am more patient with individuals from my cultural background”). Two other statements were left unchanged due to the presence of a statement with the opposite meaning in the CAS (1. " I feel uncomfortable working with families of patients from cultural backgrounds different from mine " and 2. “I usually feel less comfortable in the company of people from cultural or ethnic backgrounds different from mine “), and minor linguistic changes were made to the remaining two problematic questions to better adapt them to Polish conditions.

### Main study

#### Internal consistency

In the assessment of the CAS and its five sub-domains, Cronbach’s alpha coefficient demonstrated a high level of internal consistency, with an overall value of 0.892. Similar results were found for McDonald’s omega coefficient, which was 0.908, further validating the reliability of the instrument. Moreover, item-total correlations, analysed by correlating individual items with the overall scale score, ranged from − 0.07 to 0.69, as detailed in Table 3.

**Table 3:** Item-test correlations

Item	Mean	SD	Item-rest correlation
CA_14	5.32	1.57	0.42
CA_15	5.25	1.60	0.38
CA_16	5.20	1.62	0.38
CA_17	4.95	1.65	0.41
CA_18	4.74	1.75	0.13
CA_19	4.78	1.49	0.39
CA_20	5.69	1.40	0.46
CA_21	4.80	1.57	0.21
CA_22	5.36	1.55	0.52
CI_23*	4.15	2.04	-0.04
CI_24*	4.23	2.02	-0.07
CI_25	5.00	1.75	0.32
CI_26*	4.43	1.89	0.03
CI_27	3.76	1.72	0.18
CI_28*	4.87	1.85	0.23
CP_33	5.83	1.47	0.52
CP_34	5.75	1.47	0.52
CP_35	5.33	1.62	0.48
CP_36	5.45	1.52	0.46
GEE1	5.08	1.65	0.62
GEE2	4.97	1.56	0.62
GEE3	4.75	1.74	0.59
GEE4	4.85	1.78	0.60
GEE5*	4.54	1.88	0.25
GEE6	5.23	1.66	0.58
GEE7	5.00	1.57	0.60
GEE8	4.95	1.61	0.64
GEE9*	4.58	1.76	0.26
GEE10	4.76	1.60	0.62
GEE11	5.21	1.49	0.69
GEE12	4.78	1.47	0.63
GEE13	4.65	1.56	0.61
RI_29	4.66	1.43	0.56
RI_30	4.19	1.52	0.45
RI_31	4.67	1.44	0.60
RI_32	4.49	1.46	0.52

* Negative items

CAS domains: CA – Cognitive Awareness; CI – Behaviors/Comfort with Interactions; CP – Patients Care/Clinical Issues; GEE – General Educational Experience; RI – Research Issues

The GEE domain had the highest internal consistency (α = 0.898), whereas the CI domain had the lowest consistency (α = 0.592). A comprehensive analysis of the reliability metrics, including item-total correlations for all CAS domains, is presented in Table 4.

**Table 4:** Internal consistency analysis

CAS domains	Item	Mean	SD	Item-rest correlation	Cronbach’s α	McDonald’s ω
Cognitive Awareness (CA)	CA_14	5.32	1.57	0.63	0.778	0.789
	CA_15	5.25	1.60	0.62		
	CA_16	5.20	1.62	0.62		
	CA_17	4.95	1.65	0.43		
	CA_18	4.74	1.75	0.35		
	CA_19	4.78	1.49	0.45		
	CA_20	5.69	1.40	0.33		
	CA_21	4.80	1.57	0.42		
	CA_22	5.36	1.55	0.31		
Behaviors/Comfort with Interactions (CI)	CI_23*	4.15	2.04	0.53	0.592	0.641
	CI_24*	4.23	2.02	0.51		
	CI_25	5.00	1.75	0.27		
	CI_26*	4.43	1.89	0.49		
	CI_27	3.76	1.72	-0.17		
	CI_28*	4.87	1.85	0.39		
Patient Care/Clinical Issues (CI)	CP_33	5.83	1.47	0.60	0.791	0.795
	CP_34	5.75	1.47	0.66		
	CP_35	5.33	1.62	0.56		
	CP_36	5.45	1.52	0.59		
General Educational Experience (GEE)	GEE1	5.08	1.65	0.72	0.898	0.905
	GEE2	4.97	1.56	0.68		
	GEE3	4.75	1.74	0.65		
	GEE4	4.85	1.78	0.68		
	GEE5*	4.54	1.88	0.33		
	GEE6	5.23	1.66	0.61		
	GEE7	5.00	1.57	0.63		
	GEE8	4.95	1.61	0.68		
	GEE9*	4.58	1.76	0.32		
	GEE10	4.76	1.60	0.62		
	GEE11	5.21	1.49	0.71		
	GEE12	4.78	1.47	0.66		
	GEE13	4.65	1.56	0.63		
Research Issues (RI)	RI_29	4.66	1.43	0.61	0.815	0.817
	RI_30	4.19	1.52	0.59		
	RI_31	4.67	1.44	0.67		
	RI_32	4.49	1.46	0.67		

* Negative items

#### Theoretical validity

The suitability of the data for factor analysis was confirmed by a Kaiser‑Meyer‑Olkin (KMO) index of 0.931 (95% BCa CI 0.910–0.934) and a highly significant Bartlett’s test of sphericity (χ² = 11 583.9, df = 630, *p* < 0.001).

Using polychoric correlations, robust unweighted least‑squares (RULS) extraction coupled with Direct Oblimin rotation was applied to the full sample (*N* = 1 020). Optimal-implementation parallel analysis (PA‑polychoric) advised retention of five factors: the first five empirical eigenvalues (11.33, 3.87, 2.56, 1.80, 1.33) exceeded the 95th‑percentile random‑data benchmarks, whereas the sixth did not. Together, these five factors accounted for 58.0% of the total common variance, with the dominant first factor contributing 31.5%.

Global model fit was excellent (RMSEA = 0.045, 90% CI 0.044–0.045; CFI = 0.985; NNFI = 0.980; GFI = 0.993) and the LOSEFER‑corrected χ²/df ratio (1399.3/460 = 3.04) met recommended thresholds for ordinal data. The Solomon split‑sample procedure yielded a ratio‑communality index of 0.995, indicating equivalent subsamples and supporting the stability of the solution.

All 36 items demonstrated satisfactory sampling adequacy (MSA = 0.79–0.97); consequently, no deletions were indicated on statistical grounds. Primary pattern loadings reached |0.40| or higher for 29 of the 36 items (80.6%). The remaining seven items—CA 17,

CA 18, CA 19, CA 21, CA 22, CI 27, and CI 28—exhibited loadings in the 0.30–0.39 range but were retained because they fitted the theoretical content of their respective factors and showed acceptable communalities (≥ 0.30) with one exception noted below.

Illustrative salient loadings drawn from Table 5 include CA 15 = 0.96, CA 14 = 0.81, and CA 16 = 0.87 on Factor 3 (Cognitive Cultural Awareness); CI 23 = 0.73 and CI 24 = 0.70 on Factor 2 (Comfort with Intercultural Interactions); and CP 34 = 0.80, CP 33 = 0.70, GEE 8 = 0.81, and RI 29 = 0.69 on Factor 1 (Applied Cultural Competence in Practice). Negative or reverse‑worded items (e.g., CI 23*, GEE 5*) loaded in the expected direction, confirming the integrity of the solution.

Communalities ranged from 0.17 (CI 27) to 0.89 (CA 15), indicating that the vast majority of items shared substantial variance with the common factors. CI 27, with a communality of 0.17 and loading below|0.30|, remains the only statistical outlier and is earmarked for potential re‑wording in future iterations.

The rotated solution retained a clear simple structure: average absolute cross‑loading was 0.12, and only two items (CA 22 and CI 28) displayed cross‑loadings within 0.05 of their primary loading, well inside the pre‑specified ± 0.10 margin. Factor‑score precision indices remained robust across dimensions (ORION ≥ 0.77; Factor Determinacy ≥ 0.88; data available on request). Collectively, these results reinforce the factorial validity of the CAS and provide empirical support for the five interpretable dimensions carried forward to confirmatory modelling and substantive interpretation.

**Table 5:** Exploratory factor analysis

Item	Factor					Communality
	1	2	3	4	5	
CA_14			0.814			0.725
CA_15			0.956			0.890
CA_16			0.866			0.788
CA_17			0.321			0.307
CA_18					-0.376	0.356
CA_19	0.304					0.333
CA_20	0.474					0.405
CA_21					-0.351	0.344
CA_22	0.359	0.337				0.410
CI_23*				0.726		0.564
CI_24*				0.703		0.586
CI_25				0.413		0.306
CI_26*				0.676		0.489
CI_27						0.169
CI_28*	0.301			0.427		0.409
CP_33	0.703					0.596
CP_34	0.795					0.666
CP_35	0.654					0.507
CP_36	0.664					0.486
GEE1		0.729				0.636
GEE2		0.644				0.514
GEE3		0.515				0.661
GEE4		0.649				0.567
GEE5*		0.717				0.558
GEE6		0.768				0.592
GEE7		0.751				0.565
GEE8		0.805				0.608
GEE9*					0.629	0.474
GEE10		0.454				0.510
GEE11		0.607				0.492
GEE12		0.667				0.588
GEE13					0.644	0.511
RI_29		0.688				0.533
RI_30		0.664				0.440
RI_31		0.746				0.592
RI_32		0.657				0.502

* Negative items

CAS domains: CA – Cognitive Awareness; CI – Behaviors/Comfort with Interactions; CP – Patients Care/Clinical Issues; GEE – General Educational Experience; RI – Research Issues

#### Construct validity

The confirmatory factor analysis (CFA) results indicate that most variables demonstrate robust and statistically significant associations with their respective latent constructs (Table 6). Following refinement in the exploratory phase, six items with marginal communalities or problematic crossloadings (CA_20, CA_22, CI_25, CI_27, GEE_5, GEE_9) were removed, leaving a 30item instrument for confirmatory testing.

The ensuing fivefactor CFA—estimated on polychromic correlations and corrected with the LOSEFER scaling—reproduced the intended structure with encouraging fidelity. Although the exactfit test was significant (*χ²*(395) = 1 827, *p* < 0.001), fit indices centred on model error met accepted standards for ordinal data: SRMR = 0.067, RMSEA = 0.063 (0% CI = 0.060–0.066), CFI = 0.890, and TLI = 0.879. Residuals were small and no theoretically defensible modification indices emerged, indicating that the factor pattern required no further alteration. The results of factor covariances are shown in Table 2B (see Supplementary 2).

Within this framework every retained item aligned cleanly with its hypothesised factor and did so with meaningful strength. The cognitiveawareness dimension was anchored by CA_15, CA_14, and CA_16, whose standardised loadings exceeded 0.82. Behavioural comfort in intercultural encounters was convincingly represented by CI_24 and CI_23 (≈ 0.80). The practiceoriented factor drew solid support from CP_34 and CP_33, while the cluster of generaleducation items—most notably GEE_8—captured the reflectivehumility theme that had emerged in the exploratory stage. Finally, the researchissues factor retained its coherence, with RI_31 standing out at just over 0.80. Even the weakest loading, CI_28 at roughly 0.42, remained acceptable, and every parameter estimate exhibited narrow confidence bounds.

**Table 6:** Confirmatory factor analysis

				95% confidence interval				
Factor	Indicator	b	SE	Lower	Upper	β	Z	*p*-value
Factor 1	CA_14	1.26	0.04	1.17	1.34	0.82	29.300	< 0.001
	CA_15	1.37	0.04	1.29	1.45	0.89	32.900	< 0.001
	CA_16	1.31	0.04	1.22	1.39	0.83	29.840	< 0.001
	CA_17	0.70	0.05	0.60	0.81	0.44	13.170	< 0.001
	CA_18	0.51	0.06	0.39	0.62	0.30	8.780	< 0.001
	CA_19	0.56	0.05	0.47	0.65	0.40	12.010	< 0.001
	CA_21	0.52	0.05	0.42	0.62	0.35	10.400	< 0.001
Factor 2	CI_23*	1.58	0.06	1.46	1.70	0.80	25.640	< 0.001
	CI_24*	1.64	0.06	1.52	1.77	0.83	26.660	< 0.001
	CI_26*	1.08	0.06	0.96	1.20	0.59	17.730	< 0.001
	CI_28*	0.74	0.06	0.62	0.86	0.42	12.070	< 0.001
Factor 3	CP_33	0.95	0.04	0.87	1.03	0.74	23.350	< 0.001
	CP_34	1.06	0.04	0.98	1.15	0.78	25.150	< 0.001
	CP_35	0.99	0.05	0.89	1.09	0.64	19.610	< 0.001
	CP_36	0.93	0.05	0.83	1.02	0.64	19.630	< 0.001
Factor 4	GEE1	1.18	0.05	1.09	1.27	0.74	25.630	< 0.001
	GEE10	1.01	0.04	0.92	1.09	0.68	22.840	< 0.001
	GEE11	1.03	0.04	0.95	1.11	0.74	25.490	< 0.001
	GEE12	1.05	0.04	0.97	1.13	0.75	25.810	< 0.001
	GEE13	1.09	0.04	1.00	1.18	0.72	24.690	< 0.001
	GEE2	1.12	0.04	1.03	1.21	0.73	25.030	< 0.001
	GEE3	1.18	0.05	1.08	1.27	0.71	23.960	< 0.001
	GEE4	1.26	0.05	1.17	1.36	0.75	25.870	< 0.001
	GEE6	0.98	0.05	0.89	1.08	0.64	20.920	< 0.001
	GEE7	0.98	0.04	0.89	1.07	0.67	22.070	< 0.001
	GEE8	1.17	0.04	1.08	1.25	0.76	26.630	< 0.001
Factor 5	RI_29	1.03	0.04	0.95	1.10	0.76	25.590	< 0.001
	RI_30	0.88	0.04	0.80	0.96	0.68	22.090	< 0.001
	RI_31	1.04	0.04	0.96	1.11	0.81	28.160	< 0.001
	RI_32	0.98	0.04	0.91	1.06	0.75	25.250	< 0.001

* Negative items

b – Unstandardized coefficient, β – Standardized coefficient, SE – standard error

CAS domains: CA – Cognitive Awareness; CI – Behaviors/Comfort with Interactions; CP – Patients Care/Clinical Issues; GEE – General Educational Experience; RI – Research Issues

### Convergent validity

The convergent validity analysis demonstrated that the dimensions of Ideas (subscale of Openness to Experience), Warmth (subscale of Extraversion), and Altruism (subscale of Agreeableness) [33] showed significant correlations with all dimensions of cultural awareness as measured by the CAS. In addition, significant correlations were observed with the overall CAS score (Table 7).

**Table 7:** Correlation analysis

NEO-PI-*R* domains [26]		CA	CI	CP	GEE	RI	Total CAS
Ideas	Pearson’s r	0.176	0.306	0.300	0.159	0.082	0.280
	p-value	< 0.001	< 0.001	< 0.001	< 0.001	0.009	< 0.001
Altruism	Pearson’s r	0.251	0.193	0.401	0.307	0.161	0.387
	p-value	< 0.001	< 0.001	< 0.001	< 0.001	< 0.001	< 0.001
Warmth	Pearson’s r	0.270	0.193	0.391	0.331	0.203	0.411
	p-value	< 0.001	< 0.001	< 0.001	< 0.001	< 0.001	< 0.001

CAS domains: CA – Cognitive Awareness; CI – Behaviors/Comfort with Interactions; CP – Patients Care/Clinical Issues; GEE – General Educational Experience; RI – Research Issues

### Known-groups validity

I. ***Researcher-generated question***: *Are you open to learning about new cultures?*

Known-groups validity analysis for the question “Are you open to learning about new cultures?” revealed significant differences among groups across all five CAS scale dimensions and the total score (Table 8). One-way ANOVA results indicated that openness to new cultures has a significant impact on cultural awareness, particularly in the CI dimension (F = 25.404, *p* < 0.001, ω² = 0.046) and the overall CAS score (F = 21.191, *p* < 0.001, ω² = 0.038).

**Table 8:** The analysis of known-groups validity for the question “are you open to learning about new cultures?”

CAS domain	Yes		No		I have never thought about it		F	*P*-value*	Ω^2^
	M	SD	M	SD	M	SD			
CA	46.42	8.70	45.86	7.29	43.42	6.88	5.932	0.003	0.010
CI	27.01	6.39	22.28	6.51	23.00	5.81	25.404	< 0.001	0.046
CP	22.69	4.57	19.79	5.61	20.25	5.46	17.265	< 0.001	0.031
GEE	64.06	14.60	57.97	11.64	59.09	11.71	7.879	< 0.001	0.013
RI	18.21	4.69	17.14	4.51	16.52	4.44	6.779	0.001	0.011
CAS total	178.39	26.95	163.03	23.92	162.27	23.32	21.191	< 0.001	0.038

* One-way ANOVA

M – mean, SD – standard deviation, Ω^2^ – omega squared effect size

CAS domains: CA – Cognitive Awareness; CI – Behaviors/Comfort with Interactions; CP – Patients Care/Clinical Issues; GEE – General Educational Experience; RI – Research Issues

The Games-Howell post-hoc test revealed that individuals who were open to new cultures scored significantly higher on CI, CA, CP, GEE and RI domains than those who “had never thought about it,” indicating greater comfort and engagement with intercultural issues (Table 3C, see Supplementary 2). Specifically, significant differences in CI were found between those who were open and those who responded “No” (*p* = 0.002) or “I have never thought about it” (*p* < 0.001), suggesting that openness enhances comfort in intercultural interactions. In the CP domain, individuals who were open to new cultures also scored higher (*p* < 0.001), indicating a greater readiness to care for patients from different backgrounds.

II. ***Researcher-generated question***: *Have you ever participated in activities (e.g. lectures*,* training courses*,* workshops*,* exercises*,* seminars) on intercultural communication?*

The analysis of known-groups validity revealed significant differences in the level of cultural awareness between nursing students who had participated in intercultural communication courses and those who either had no such experience or could not recall it (Table 9). A one-way ANOVA revealed significant differences among the groups across all dimensions of the CAS. The most pronounced differences were observed in the dimensions of CA (F = 15.460, *p* < 0.001) and GEE (F = 32.882, *p* < 0.001, Ω² = 0.063, respectively). Significant differences were also found in the RI dimension (F = 35.510, *p* < 0.001, Ω² = 0.068) as well as in the overall CAS score, with participants in the intercultural communication courses achieving higher scores (F = 29.266, *p* < 0.001, Ω² = 0.055).

**Table 9:** The analysis of known-groups validity for the question: “have you ever participated in activities (e.g. Lectures, training courses, workshops, exercises, seminars) on intercultural communication?”

CAS domain	Yes		No		I don’t know / I don’t remember		F	*P*-value*	Ω^2^
	M	SD	M	SD	M	SD			
CA	47.77	8.44	45.14	8.44	44.24	8.30	15.460	< 0.001	0.028
CI	26.47	6.48	26.90	6.66	25.41	6.01	3.358	0.035	0.005
CP	22.97	4.55	22.13	4.72	21.41	5.23	7.571	0.001	0.013
GEE	67.03	14.00	59.26	14.44	63.99	12.43	32.882	< 0.001	0.063
RI	19.38	4.69	16.79	4.63	17.49	3.86	35.510	< 0.001	0.068
CAS total	183.60	26.39	170.23	26.72	172.54	25.22	29.266	< 0.001	0.055

* One-way ANOVA

M – mean, SD – standard deviation, Ω^2^ – omega squared effect size

CAS domains: CA – Cognitive Awareness; CI – Behaviors/Comfort with Interactions; CP – Patients Care/Clinical Issues; GEE – General Educational Experience; RI – Research Issues

Further analysis using the Games-Howell post-hoc test confirmed the observed differences between the groups (Table 4D, see Supplementary 2). In the GEE dimension, the mean difference was − 7.77 (*p* < 0.001) between students who attended intercultural communication courses and those who had never participated in such courses, emphasizing the impact of intercultural education on the perception of intercultural diversity. Similarly, in the RI dimension, the difference was − 2.58 (*p* < 0.001), indicating a higher research awareness among participants of these courses.

## Discussion

The results of our study confirm that the undertaken cultural and linguistic adaptation was adequate, as reflected in the high internal consistency and accuracy of the scale. The Cronbach’s alpha coefficient for the entire scale was 0.892, which indicates a high level of reliability of the tool in the Polish context and confirms its stability and reliability in assessing cultural competence. South Korea and Cyprus researchers obtained similar results (0.89 and 0.86, respectively) [12, 13]. This figure is also close to the results obtained in the original CAS studies, where the coefficient was 0.82 [10]. The difference may be due to linguistic adaptation and cultural differences between the American population, for whom the scale was originally developed, and Polish nursing students, reflecting the effectiveness of the translation and adaptation process. The high reliability of the scale in its Polish version shows that the design of the tool is universal enough to retain its consistency and to measure the same aspects of cultural competence, despite cultural differences.

In addition, the high Cronbach’s alpha coefficient shows that the individual scale items correlate well with each other, indicating that the scale comprehensively measures the different dimensions of cultural awareness. Importantly, the cultural adaptation did not compromise the original theoretical constructs of the scale, a key objective of this study. Linguistic adaptation involved not only translation itself, but it also accounted for the cultural context of Polish nursing students. This process required close collaboration with experts in various scientific fields to ensure that each item retained its original meaning while being clear and appropriate for Polish respondents. The results of the psychometric analysis, including the high reliability coefficient, suggest that this step was carried out effectively.

On the other hand, the lower value of the internal consistency coefficient for Behaviors/Comfort with Interactions (CI) suggests that Polish nursing students may perceive intercultural interactions differently. The Cronbach’s alpha coefficient of 0.592 is noticeably lower compared to the other CAS_P subscales, which may indicate that students have different perceptions and behaviors regarding their comfort in interacting with people from different cultural backgrounds. This may be related to several factors, including differences in education, the degree of exposure to cultural diversity, and the extent of development of cultural competence in Polish tertiary nursing education. Interestingly, researchers from South Korea and the author of the scale also obtained low internal consistency coefficient values for Behaviors/Comfort with Interactions (CI) [10, 12]. What is of note is that the lower consistency of this subscale may reflect not only the different attitudes of students towards intercultural interactions, but also the peculiarities of Polish culture, in which issues of cultural diversity may be given less emphasis in medical education than in countries such as the United States. Less exposure to cultural diversity in the daily and professional lives of nursing students may affect their perceived comfort in interacting with people from different cultures, which may have contributed to lower cohesion index scores [6].

There are also specific components of educational programs that may affect the level of comfort in intercultural interactions. Cultural competence training in the Polish context may not be comprehensive enough to effectively prepare students to work in multicultural settings. The low internal consistency of the CI subscale may indicate that students are not provided with enough practical tools and hands-on experiences to help them feel more confident in situations involving interactions with people from different cultures. This may also be a result of the limited number of clinical scenarios in which students have the opportunity to work with patients from different cultural backgrounds, which in turn may increase their sense of comfort in such interactions [7].

The results of the construct validity analysis of CAS_P, based on exploratory factor analysis (EFA) and confirmatory factor analysis (CFA), confirm the multidimensional structure of the research tool, but indicate a moderate model fit. A CFI of 0.797 and a TLI of 0.781 suggest that the model largely accounts for the variables covered by the scale. However, it does not fully meet the criteria for a perfect fit. These values indicate some limitations, which may be due to cross-cultural differences or to the specifics of the Polish nursing education and highlight the need for further analysis of the adapted scale. Similar, although slightly better, construct validity results were obtained by researchers from Cyprus, suggesting a better model fit with that population [13].

The highest factor loadings were obtained for variables related to General Educational Experience (GEE), confirming the important role of education in the development of nursing students’ cultural awareness. These findings suggest that educational programs should place more emphasis on cultural diversity in order to effectively promote the development of intercultural competence in future healthcare professionals. Higher model fit values for dimensions such as the GEE indicate that education is one of the most important factors contributing to the development of cultural competence. This highlights the need for the further development and integration of such content into the curricula of nursing education programs.

Apart from the key role of education, the results of the CFA analysis suggest that other dimensions of the scale, such as Cognitive Awareness (CA) and Patient Care/Clinical Issues (CI), also have high factor loadings, although not as evident as the GEE. This indicates the multidimensional nature of cultural competence, which is not limited to theoretical education, but also involves an understanding of the impact of culture on one’s beliefs and attitudes, as well as having the practical skills needed to provide culturally competent care. This implies that educational programs should focus not only on the provision of theoretical knowledge, but also on the development of practical skills and attitudes necessary for working in culturally diverse clinical settings [7, 35].

As for Behaviors/Comfort with Interactions (CI), lower scores suggest that Polish students may be uncomfortable with intercultural interactions, which may be due to specific local socio-cultural determinants. This suggests the need for further modifications to this dimension of the scale in order to better reflect the influence of various factors on the differences in students’ attitudes and experiences in the Polish context. In order for students to become more confident in working with patients from different cultural backgrounds, the introduction of more in-depth educational content and practical learning activities may be required.

The results of the convergent validity analysis suggest that personality traits such as altruism, warmth and openness to new ideas are correlate with certain aspects of cultural awareness in nursing education, particularly comfort with cross-cultural interactions and intercultural competence in patient care. As previous research has shown, pro-social attitudes, such as altruism, may promote empathy with patients from different cultural backgrounds, which is crucial in the context of culturally competent care [36–38]. The correlation between warmth and the cognitive awareness of cultural diversity is supported by research showing that a high degree of openness and empathy are key to understanding the impact of culture on patient health and behavior [39, 40]. These correlations support the validity of using the CAS to assess cultural awareness and suggest that individual personality traits may influence different aspects of cultural competence, which has important implications for nursing education.

The results of the known-groups validity analysis confirmed that reported openness to learning about new cultures and participation in intercultural communication courses were significantly associated with increased levels of cultural awareness as measured by the Cultural Awareness Scale (CAS) in nursing students. The study showed that respondents who reported openness to new cultures scored higher on comfort with intercultural interactions (CI). This is consistent with the findings of Sparkman et al., who demonstrated that openness and willingness to learn about other cultures have a positive effect on interpersonal skills in an intercultural context [41]. Participation in intercultural communication courses was also found to be strongly associated with higher scores for General Educational Experience (GEE). This highlights the role of formal intercultural education in developing cultural competence and is consistent with previous literature reports [42]. These findings support the concept that education and personal engagement in learning about cultural diversity can effectively promote the development of cultural awareness, which is particularly important for future health professionals [43].

A final aspect of the CAS_P validation process worth highlighting is the exclusion of one item from the final Polish version of the scale. Following a pilot study and expert review, we decided to delete the item ‘My nursing instructors seem interested in learning how their classroom behaviour may discourage students from certain cultural or ethnic groups’. In the Polish cultural context, this sentence could cause interpretation difficulties for students due to different views on issues related to teacher behavior and student-teacher relationships. The validity and reliability of this item could be compromised by a lack of direct experience with or understanding of ethnic diversity among nursing students, potentially resulting in inaccurate assessments of the impact of teachers’ behavior on ethnic minority students.

### Limitations

Despite the positive validation results of the CAS_P scale, some limitations of the survey are worth mentioning. First, the Behaviours/Comfort with Interactions (CI) subscale is only sufficiently accurate and requires further adaptation in a larger group of students to better reflect the specifics of the Polish cultural context. The other limitation is the possibility of self-report errors, as the CAS scale is based on subjective reports of the respondents, which may not reflect the actual level of cultural competence. Another limitation is the incomplete control of external variables, such as students’ previous work experience in a multicultural setting or contact with people from other cultures.

### Practical implications

Further research can help to optimize tools for assessing cultural competence and to better adapt nursing education programs to the needs of a changing society. These findings can inform the development of educational policies and training programs that foster cultural competence, not only in nursing but also in other allied health professions. Future research should explore the impact of additional variables, such as international experiences and personal culture-related activities, on the development of cultural competence.

The English language version of the validated CAS can be found in Supplementary 3 and Polish version of CAS_P can be found in Supplementary 4.

## Conclusions

The Polish version of the Cultural Awareness Scale (CAS_P) is a psychometrically sound instrument for assessing cultural awareness among nursing students. Its reliability, confirmed through internal consistency measures, and its validity, supported by factor analysis and external correlations, demonstrate that it can be effectively used in the Polish educational context.

The tool provides a foundation for systematically evaluating students’ readiness to deliver culturally appropriate care in an increasingly diverse society. Its use can support both individual assessment and institutional curriculum planning in nursing education. While the Behaviors/Comfort with Interactions subscale revealed some cultural sensitivity issues, this highlights the value of further refinement and adaptation to better reflect local sociocultural conditions. Overall, the CAS_P fills an important gap in Polish nursing education by offering an evidence-based measure for cultural competence development.

## Electronic supplementary material

Below is the link to the electronic supplementary material.

Supplementary Material 1

Supplementary Material 2

Supplementary Material 3

Supplementary Material 4

## Data Availability

The data sets used for the current study are available from the corresponding author upon request.

## Abbreviations

CAS: Cultural Awareness Scale
IP: Internet Protocol
WHO: World Health Organization
