# Supplementary material for: Cultural awareness scale: psychometric properties and applicability in assessing cultural competence among polish nursing students
Source: BMC Nurs. 2025 May 15;24:542. doi: 10.1186/s12912-025-03181-y (PMC12082968; doi:10.1186/s12912-025-03181-y)
Supplement: Supplementary file 1 — Supplementary Material 1 [file 12912_2025_3181_MOESM1_ESM.docx]

**1. In your opinion, were the questions clear and easy to understand?**

*(Did you have any difficulty understanding any of the questions?)*

**2. How would you rate the length of the survey?**

*(Was it appropriately short, long or did it take too long?)*

**3. What were your first thoughts after completing the survey?**

*(Did you feel satisfied, relieved or perhaps frustrated?)*

**4. Do you feel that taking part in the survey can have an impact on improving nursing education?**

*(Do you think that your contribution can make a difference?)*

**5. Did you feel comfortable answering questions about cultural differences?**

*(Did you find it easy to discuss the topic of cultural awareness?)*

**6. In your opinion, were the questions appropriate for the Polish cultural context?**

*(Were the questions consistent with your experiences in the Polish educational and professional setting?)*

**7. Did you feel any pressure to complete the questionnaire?**

*(Did you answer under time pressure or pressure to meet the expectations of others?)*

8. **Would you recommend to other students to participate in similar surveys?** **Why yes/no?**

*(What is your overall impression of participating in this survey?)*

9. **Were all the words used in the survey clear to you?**

*(Were there any terms or phrases that were difficult to understand?)*

10. **How would you rate the choice of vocabulary in the survey items?**

*(Was the language of the survey formal, colloquial or appropriate?)*

11. **Did you come across any words that needed further explanation?**

*(Which ones and why?)*

12. **Were the questions worded in unambiguously?**

*(Were there any phrases that could be interpreted in different ways?)*

13. **Were there any phrases or technical terms in the survey that were not clear to you?**

*(How could these be simplified or clarified?)*

14. **Did the language of the survey seem appropriate to the Polish cultural context?**

*(Did any words or phrases seem unnatural or foreign to you?)*

15. **Which questions seemed most complicated in terms of language?**

*(What made them difficult?)*

16. **Was the grammatical structure of the questions clear?**

*(Was the wording too complex or unclear?)*

17. **In your opinion, was the use of negation in the survey items clear?**

*(Were the negation questions easy to understand?)*

18. **What changes to the lexicon of the survey could improve its comprehensibility?**

*(Do you see a need to simplify or standardise certain expressions?)*

19. **Do you think that the vocabulary used in the survey items was culturally neutral?**

*(Were there any phrases that might be considered inappropriate by people from different backgrounds?)*
