# Supplementary material for: Cultural awareness scale: psychometric properties and applicability in assessing cultural competence among polish nursing students
Source: BMC Nurs. 2025 May 15;24:542. doi: 10.1186/s12912-025-03181-y (PMC12082968; doi:10.1186/s12912-025-03181-y)
Supplement: Supplementary file 2 — Supplementary Material 2 [file 12912_2025_3181_MOESM2_ESM.docx]

Table 1A. Item-test correlations (pilot study)

| **Item** | **Mean** | **SD** | **Item-rest correlation** |
| --- | --- | --- | --- |
| 1. Lecturers at the nursing school where I study adequately address the problem of multiculturalism in nursing | 4.94 | 1.63 | 0.49822 |
| 1. The nursing school where I study provides nursing students with opportunities to undertake activities related to multiculturalism | 4.67 | 1.66 | 0.48380 |
| 1. Since starting nursing studies at this school, my understanding of issues related to multiculturalism has increased | 4.40 | 1.82 | 0.50114 |
| 1. The experience gained during my studies in nursing at this school has helped me gain knowledge about health problems of various ethnic and cultural groups | 4.35 | 1.89 | 0.59384 |
| 1. I think my cultural identity influences my attitudes | 5.03 | 1.76 | 0.37735 |
| 1. I think my cultural identity influences my beliefs | 4.85 | 1.73 | 0.46123 |
| 1. I think my cultural identity influences my behavior | 2.34 | 1.80 | 0.20336 |
| 1. I often wonder how culture influences people's beliefs, attitudes, and behaviors | 4.74 | 2.02 | 0.28144 |
| 1. I feel comfortable working with patients of all ethnic groups | 4.85 | 1.58 | 0.27218 |
| 1. I believe that nurses' cultural beliefs influence their nursing care decisions | 2.95 | 1.79 | 0.20521 |
| 1. I have noticed that lecturers at my nursing school turn to students from cultural minorities when issues related to their ethnic group come up in class | 3.56 | 1.89 | 0.38427 |
| 1. I have noticed that lecturers in the nursing school make every effort to ensure that no student is excluded during group discussions or exercises | 5.13 | 1.72 | 0.41461 |
| 1. I believe that the cultural values of students influence their behavior during classes (e.g. asking questions, participating in group activities or commenting). | 4.71 | 1.68 | 0.46792 |
| 1. I believe that it is the responsibility of nursing lecturers to accommodate diverse educational needs of students | **3.01** | **1.72** | **0.00957** |
| 1. Lecturers at my nursing school feel comfortable when discussing cultural issues during classes | 5.84 | 1.55 | 0.43599 |
| 1. I believe that the cultural values of lecturers influence their behavior during clinical practice | 5.10 | 1.50 | 0.60657 |
| 1. I believe that the experience gained when studying at the nursing school helps students feel more comfortable interacting with people from different cultures | 4.12 | 1.67 | 0.57650 |
| 1. I feel comfortable discussing cultural issues during classes | 4.70 | 1.83 | 0.39363 |
| 1. Clinical classes carried out at my nursing school have helped me feel more comfortable when interacting with people from different cultures | 4.67 | 1.48 | 0.55791 |
| 1. I believe that lecturers at my nursing school respect the differences between people from different cultural backgrounds | 3.37 | 1.67 | 0.22309 |
| 1. Lecturers at my nursing school model behaviors that demonstrate sensitivity to muticulturalism-related issues | 5.20 | 1.48 | 0.51960 |
| 1. Lecturers at my nursing school use examples and/or case studies that comprise information concerning different cultural and ethnic groups | 4.33 | 1.73 | 0.45274 |
| 1. Research and teaching staff at my nursing school conduct research concerning the multicultural aspect of health issues | 4.00 | 1.66 | 0.46080 |
| 1. Students at my nursing school prepared theses on cultural differences related to health issues | 4.39 | 1.54 | 0.50884 |
| 1. The faculty at my nursing school takes account of the significance of different data in relation to the studied cultural groups | 4.28 | 1.51 | 0.48837 |
| 1. Research and teaching staff at my nursing school take cultural issues into account when interpreting research outcomes | 5.53 | 1.81 | 0.32311 |
| 1. I respect my patients' decisions related to their culture, even if I disagree with them | 5.78 | 1.36 | 0.41934 |
| 1. If I needed more information about a patient's culture, I would use available resources (e.g., books, movies) | 4.99 | 1.74 | 0.35959 |
| 1. If I needed more information about a patient's culture, I would not hesitate to ask my colleagues about it | 5.43 | 1.41 | 0.21939 |
| 1. If I needed more information about a patient's culture, I would not hesitate to ask the patient or a person from her/his family about it | 2.95 | 1.84 | 0.33290 |
| 1. When I have an opportunity to help someone, I am less likely to offer help to people from other cultural backgrounds* | **2.76** | **1.68** | **-0.54577** |
| 1. I am less patient with people from other cultural backgrounds* | **3.36** | **1.81** | **-0.51424** |
| 1. I usually feel less comfortable in the company of people from cultural or ethnic backgrounds different from mine* | **5.46** | **1.88** | **-0.21609** |
| 1. During nursing studies, my lecturers exhibited behaviors that could make students from some cultural backgrounds feel excluded* | **2.44** | **1.41** | **-0.37956** |
| 1. Lecturers at my nursing school seem interested in finding out how their behavior during classes may discourage students from certain cultural or ethnic groups* | **3.55** | **1.81** | **-0.37416** |
| 1. I believe that certain aspects of teaching at the nursing school where I study may alienate students from some cultural backgrounds* | **3.41** | **1.56** | **-0.58125** |
| 1. I feel uncomfortable working with families of patients from cultural backgrounds different from mine* | **3.40** | **1.60** | **-0.53165** |

*Reverse-coded items

Table 2B. Factor Covariances

|  | | | | **95% Confidence Interval** | |  |  | |
| --- | --- | --- | --- | --- | --- | --- | --- | --- |
|  |  | **b** | **SE** | **Lower** | **Upper** | **β** | **Z** | **p-value** |
| CA | CA | 1.00 |  |  |  |  |  |  |
|  | CI | -0.36 | 0.03 | -0.42 | -0.29 | -0.36 | -10.850 | < .001 |
|  | CP | 0.34 | 0.03 | 0.27 | 0.41 | 0.34 | 10.050 | < .001 |
|  | GEE | 0.42 | 0.03 | 0.36 | 0.47 | 0.42 | 14.200 | < .001 |
|  | RI | 0.40 | 0.03 | 0.34 | 0.46 | 0.40 | 12.680 | < .001 |
| CI | CI | 1.00 |  |  |  |  |  |  |
|  | CP | 0.11 | 0.04 | 0.04 | 0.19 | 0.11 | 2.870 | 0.004 |
|  | GEE | -0.15 | 0.04 | -0.22 | -0.08 | -0.15 | -4.270 | < .001 |
|  | RI | -0.29 | 0.04 | -0.36 | -0.22 | -0.29 | -8.260 | < .001 |
| CP | CP | 1.00 |  |  |  |  |  |  |
|  | GEE | 0.51 | 0.03 | 0.46 | 0.57 | 0.51 | 17.730 | < .001 |
|  | RI | 0.46 | 0.03 | 0.40 | 0.53 | 0.46 | 14.380 | < .001 |
| GEE | GEE | 1.00 |  |  |  |  |  |  |
|  | RI | 0.83 | 0.02 | 0.80 | 0.86 | 0.83 | 53.850 | < .001 |
| RI | RI | 1.00 |  |  |  |  |  |  |

b – Unstandardized coefficient, β – Standardized coefficient, SE – standard error

CAS domains: CA – Cognitive Awareness; CI – Behaviors/Comfort with Interactions; CP – Patients Care/Clinical Issues; GEE – General Educational Experience; RI – Research Issues

Table 3C. The analysis of The Games-Howell post-hoc test for the item "Are you open to learning about new cultures?"

| **CAS domain** | **Possible answers** | **Games-Howell Post-Hoc Test** | | | |
| --- | --- | --- | --- | --- | --- |
|  |  |  | **Yes** | **No** | **I have never thought about that** |
| CA | **Yes** | Mean difference | - | 0.557 | 3.00 |
|  |  | p-value | - | 0.915 | **<.001** |
|  | **No** | Mean difference |  | - | 2.45 |
|  |  | p-value |  | - | 0.248 |
|  | **I have never thought about that** | Mean difference |  |  | - |
|  |  | p-value |  |  | - |
| CI | **Yes** | Mean difference | - | 4.73 | 4.006 |
|  |  | p-value | - | 0.002 | **<.001** |
|  | **No** | Mean difference |  | - | -0.724 |
|  |  | p-value |  | - | 0.851 |
|  | **I have never thought about that** | Mean difference |  |  | - |
|  |  | p-value |  |  | - |
| GEE | **Yes** | Mean difference | - | 6.09 | 4.96 |
|  |  | p-value | - | 0.026 | **<.001** |
|  | **No** | Mean difference |  | - | -1.13 |
|  |  | p-value |  | - | 0.889 |
|  | **I have never thought about that** | Mean difference |  |  | - |
|  |  | p-value |  |  | - |
| CP | **Yes** | Mean difference | - | 2.90 | 2.447 |
|  |  | p-value | - | 0.026 | **<.001** |
|  | **No** | Mean difference |  | - | -0.452 |
|  |  | p-value |  | - | 0.921 |
|  | **I have never thought about that** | Mean difference |  |  | - |
|  |  | p-value |  |  | - |
| RI | **Yes** | Mean difference | - | 1.07 | 1.694 |
|  |  | p-value | - | 0.428 | **<.001** |
|  | **No** | Mean difference |  | - | 0.619 |
|  |  | p-value |  | - | 0.789 |
|  | **I have never thought about that** | Mean difference |  |  | - |
|  |  | p-value |  |  | - |
| CAS total | **Yes** | Mean difference | - | 15.4 | 16.113 |
|  |  | p-value | - | 0.005 | **<.001** |
|  | **No** | Mean difference |  |  | 0.761 |
|  |  | p-value |  |  | 0.987 |
|  | **I have never thought about that** | Mean difference |  |  | - |
|  |  | p-value |  |  | - |

CAS domains: CA – Cognitive Awareness; CI – Behaviors/Comfort with Interactions; CP – Patients Care/Clinical Issues; GEE – General Educational Experience; RI – Research Issues

Table 4D. The analysis of The Games-Howell post-hoc test for the item: „Have you ever participated in activities (e.g. lectures, training courses, exercises, seminars) on intercultural communication?”

| **CAS domain** | **Possible answers** | **Games-Howell Post-Hoc Test** | | | |
| --- | --- | --- | --- | --- | --- |
|  |  |  | **Yes** | **No** | **I don’t know/I don’t remember** |
| CA | **Yes** | Mean difference | - |  |  |
|  |  | p-value | - |  |  |
|  | **No** | Mean difference | -2.62 | - | 0.899 |
|  |  | p-value | <0.001 | - | 0.451 |
|  | **I don’t know/I don’t remember** | Mean difference | -3.52 |  | - |
|  |  | p-value | <0.001 |  | - |
| CI | **Yes** | Mean difference | - |  |  |
|  |  | p-value | - |  |  |
|  | **No** | Mean difference | 0.440 | - | 1.50 |
|  |  | p-value | 0.596 | - | 0.020 |
|  | **I don’t know/I don’t remember** | Mean difference | -1.060 |  | - |
|  |  | p-value | 0.129 |  | - |
| GEE | **Yes** | Mean difference | - |  |  |
|  |  | p-value | - |  |  |
|  | **No** | Mean difference | -7.77 |  | -4.73 |
|  |  | p-value | <.001 |  | <.001 |
|  | **I don’t know/I don’t remember** | Mean difference | -3.04 |  | - |
|  |  | p-value | 0.023 |  | - |
| CP | **Yes** | Mean difference | - |  |  |
|  |  | p-value | - |  |  |
|  | **No** | Mean difference | -0.833 | - | 1.50 |
|  |  | p-value | 0.025 | - | 0.020 |
|  | **I don’t know/I don’t remember** | Mean difference | -1.554 |  | - |
|  |  | p-value | 0.002 |  | - |
| RI | **Yes** | Mean difference | - |  |  |
|  |  | p-value | - |  |  |
|  | **No** | Mean difference | -2.58 |  | -0.702 |
|  |  | p-value | <.001 |  | 0.138 |
|  | **I don’t know/I don’t remember** | Mean difference | -1.88 |  | - |
|  |  | p-value | <0.001 |  | - |
| CAS total | **Yes** | Mean difference | - |  |  |
|  |  | p-value | - |  |  |
|  | **No** | Mean difference | -13.4 | - | -2.31 |
|  |  | p-value | <.001 | - | 0.573 |
|  | **I don’t know/I don’t remember** | Mean difference | -11.1 |  | - |
|  |  | p-value | <0.001 |  | - |

CAS domains: CA – Cognitive Awareness; CI – Behaviors/Comfort with Interactions; CP – Patients Care/Clinical Issues; GEE – General Educational Experience; RI – Research Issues
