# Supplementary material for: Cultural awareness scale: psychometric properties and applicability in assessing cultural competence among polish nursing students
Source: BMC Nurs. 2025 May 15;24:542. doi: 10.1186/s12912-025-03181-y (PMC12082968; doi:10.1186/s12912-025-03181-y)
Supplement: Supplementary file 3 — Supplementary Material 3 [file 12912_2025_3181_MOESM3_ESM.docx]

**CULTURAL AWARENESS SCALE (CAS_P)**

| Lecturers at the nursing school where I study adequately address the problem of multiculturalism in nursing |
| --- |
| The nursing school where I study provides nursing students with opportunities to undertake activities related to multiculturalism |
| Since starting nursing studies at this school, my understanding of issues related to multiculturalism has increased |
| The experience gained during my studies in nursing at this school has helped me gain knowledge about health problems of various ethnic and cultural groups |
| I think my cultural identity influences my attitudes |
| I think my cultural identity influences my beliefs |
| I think my cultural identity influences my behavior |
| I often wonder how culture influences people's beliefs, attitudes, and behaviors |
| When I have an opportunity to help someone, I am more likely to offer help to people from my cultural background |
| I am more patient with people from my cultural background* |
| I feel comfortable working with patients of all ethnic groups* |
| I believe that nurses' cultural beliefs influence their nursing care decisions |
| I usually feel less comfortable in the company of people from cultural or ethnic backgrounds different from mine* |
| I have noticed that lecturers at my nursing school turn to students from cultural minorities when issues related to their ethnic group come up in class |
| I have noticed that lecturers in the nursing school make every effort to ensure that no student is excluded during group discussions or exercises |
| I believe that the cultural values of students influence their behavior during classes (e.g. asking questions, participating in group activities or commenting). |
| During nursing studies, my lecturers exhibited behaviors that could make students from some cultural backgrounds feel excluded* |
| I believe that it is the responsibility of nursing lecturers to accommodate diverse educational needs of students |
| Lecturers at my nursing school feel comfortable when discussing cultural issues during classes |
| I believe that the cultural values of lecturers influence their behavior during clinical practice |
| I believe that the experience gained when studying at the nursing school helps students feel more comfortable interacting with people from different cultures |
| I believe that certain aspects of teaching at the nursing school where I study may alienate students from some cultural backgrounds* |
| I feel comfortable discussing cultural issues during classes |
| Clinical classes carried out at my nursing school have helped me feel more comfortable when interacting with people from different cultures |
| I believe that lecturers at my nursing school respect the differences between people from different cultural backgrounds |
| Lecturers at my nursing school model behaviors that demonstrate sensitivity to muticulturalism-related issues |
| Lecturers at my nursing school use examples and/or case studies that comprise information concerning different cultural and ethnic groups |
| Research and teaching staff at my nursing school conduct research concerning the multicultural aspect of health issues |
| Students at my nursing school prepared theses on cultural differences related to health issues |
| The faculty at my nursing school takes account of the significance of different data in relation to the studied cultural groups |
| Research and teaching staff at my nursing school take cultural issues into account when interpreting research outcomes |
| I respect my patients' decisions related to their culture, even if I disagree with them |
| If I needed more information about a patient's culture, I would use available resources (e.g., books, movies) |
| If I needed more information about a patient's culture, I would not hesitate to ask my colleagues about it |
| If I needed more information about a patient's culture, I would not hesitate to ask the patient or a person from her/his family about it |
| I feel uncomfortable working with families of patients from cultural backgrounds different from mine* |

*Negative items
