# Supplementary material for: Cultural awareness scale: psychometric properties and applicability in assessing cultural competence among polish nursing students
Source: BMC Nurs. 2025 May 15;24:542. doi: 10.1186/s12912-025-03181-y (PMC12082968; doi:10.1186/s12912-025-03181-y)
Supplement: Supplementary file 4 — Supplementary Material 4 [file 12912_2025_3181_MOESM4_ESM.docx]

**SKALA ŚWIADOMOŚCI KULTUROWEJ (CAS_P)**

| Wykładowcy w uczelni, w której zdobywam wykształcenie pielęgniarskie, odpowiednio odnoszą się do problemu wielokulturowości w pielęgniarstwie |
| --- |
| Uczelnia w której zdobywam wykształcenie pielęgniarskie, umożliwia studentkom/studentom pielęgniarstwa podejmowanie działań związanych z wielokulturowością |
| Od momentu rozpoczęcia studiów pielęgniarskich w tej uczelni wzrosło moje zrozumienie dla zagadnień związanych z wielokulturowością |
| Doświadczenia wyniesione w trakcie studiów na kierunku pielęgniarstwo w tej uczelni pomogły mi zdobyć wiedzę na temat problemów zdrowotnych różnych grup etnicznych i kulturowych |
| Myślę, że moja przynależność kulturowa wpływa na moje postawy |
| Myślę, że moja przynależność kulturowa wpływa na moje przekonania |
| Myślę, że moja przynależność kulturowa wpływa na moje zachowanie |
| Często zastanawiam się nad tym, jak kultura wpływa na przekonania, postawy i zachowania ludzi |
| Kiedy mam możliwość komuś pomóc, częściej oferuję pomoc osobom z mojego kręgu kulturowego* |
| Jestem bardziej cierpliwy wobec osób z mojego kręgu kulturowego* |
| Czuję się komfortowo pracując z pacjentkami/pacjentami pochodzącymi ze wszystkich grup etnicznych |
| Uważam, że przekonania kulturowe pielęgniarek wpływają na ich decyzje dotyczące opieki pielęgniarskiej |
| Zazwyczaj czuję się mniej komfortowo w towarzystwie osób o innym niż moje pochodzeniu kulturowym lub etnicznym* |
| Zauważyłam/Zauważyłem, że wykładowcy w uczelni, w której zdobywam wykształcenie pielęgniarskie, zwracają się do studentek/studentów pochodzących z mniejszości kulturowych, gdy na zajęciach pojawiają się kwestie związane z ich grupą etniczną |
| Zauważyłam/Zauważyłem, że wykładowcy na kierunku pielęgniarstwo dokładają starań, aby żadna studentka/żaden student nie był wykluczony podczas dyskusji w grupie lub w czasie ćwiczeń |
| Uważam, że wartości kulturowe studentek/studentów wpływają na ich zachowania w trakcie zajęć dydaktycznych (np. zadawanie pytań, podejmowanie aktywności w grupach lub komentowanie) |
| Podczas studiów pielęgniarskich zachowania niektórych wykładowców mogły prowadzić do poczucia wykluczenia wśród studentek/studentów pochodzących z różnorodnych środowisk kulturowych* |
| Uważam, że obowiązkiem wykładowcy na kierunku pielęgniarstwo jest uwzględnienie różnorodnych potrzeb edukacyjnych studentek/studentów |
| Wykładowcy na studiach pielęgniarskich w uczelni w której studiuję, ze swobodą omawiają kwestie kulturowe podczas zajęć dydaktycznych |
| Uważam, że wartości kulturowe wykładowców wpływają na ich zachowania podczas praktyki klinicznej |
| Uważam, że doświadczenia zdobyte podczas studiów pielęgniarskich w uczelni, w której studiuję pomagają studentkom/studentom poczuć się bardziej komfortowo w kontaktach z osobami z różnych kultur |
| Niektóre metody dydaktyczne w mojej uczelni sprawiają, że studentki/ studenci z różnych kultur mogą poczuć się marginalizowani* |
| Czuję się komfortowo uczestnicząc w omawianiu kwestii kulturowych podczas zajęć dydaktycznych |
| Zajęcia kliniczne realizowane w uczelni, w której zdobywam wykształcenie pielęgniarskie, pomogły mi poczuć się bardziej komfortowo w kontaktach z osobami pochodzącymi z różnych kultur |
| Uważam, że wykładowcy na studiach pielęgniarskich w uczelni, w której studiuję szanują różnice występujące między osobami pochodzącymi z różnych kręgów kulturowych |
| Wykładowcy w uczelni, w której zdobywam wykształcenie pielęgniarskie, modelują zachowania świadczące o wrażliwości na kwestie związane z wielokulturowością |
| Wykładowcy na studiach pielęgniarskich w uczelni, w której studiuję, wykorzystują przykłady i/lub studia przypadków, które uwzględniają informacje dotyczące różnych grup kulturowych i etnicznych |
| Pracownicy naukowi i dydaktyczni w uczelni, w której zdobywam wykształcenie pielęgniarskie, prowadzą badania uwzględniające zagadnienia zdrowotne w aspekcie wielokulturowym |
| Studentki/Studenci w uczelni, w której zdobywam wykształcenie pielęgniarskie, przygotowywali prace dyplomowe, które dotyczyły różnic kulturowych związanych z kwestiami zdrowotnymi |
| Kadra akademicka w uczelni, w której zdobywam wykształcenie pielęgniarskie, bierze pod uwagę znaczenie różnych danych w odniesieniu do badanych grup kulturowych |
| Pracownicy naukowi i dydaktyczni w uczelni, w której zdobywam wykształcenie pielęgniarskie, uwzględniają kwestie kulturowe podczas interpretacji wyników swoich badań |
| Szanuję decyzje moich pacjentek/pacjentów mające związek z ich kulturą, nawet jeśli się z nimi nie zgadzam |
| Gdybym potrzebowała/potrzebował więcej informacji na temat kultury z której pochodzi pacjentka/ pacjent, skorzystałabym/skorzystałbym z istniejących zasobów (na przykład książek, filmów itp.) |
| Gdybym potrzebowała/potrzebował więcej informacji na temat kultury z której pochodzi pacjentka/pacjent, bez wahania zapytałabym/zapytałbym o to moich współpracowników |
| Gdybym potrzebowała/potrzebował więcej informacji na temat kultury z której pochodzi pacjentka/ pacjent, bez wahania zapytałabym/zapytałbym o to samej pacjentki/samego pacjenta lub osoby z jej/jego rodziny |
| Czuję się niekomfortowo pracując z rodzinami pacjentek lub pacjentów z kręgów kulturowych odmiennych niż mój* |

*Pytania z odwróconą punktacją
